# Assessment of PTSD in military personnel via machine learning based on physiological habituation in a virtual immersive environment

**DOI:** 10.1038/s41598-025-91916-x

**Published:** 2025-03-04

**Authors:** Gauthier Pellegrin, Nicolas Ricka, Denis A. Fompeyrine, Thomas Rohaly, Leah Enders, Heather Roy

**Affiliations:** 1 MyndBlue, Paris, F-75008 France; 2 DCS Corporation, Alexandria, Virginia United States of America; 3 Human in Complex Systems Division, DEVCOM Army Research Laboratory, Aberdeen Proving Ground, Maryland United States of America

**Keywords:** Posttraumatic Stress Disorder, Artificial intelligence, Machine Learning, Precision psychiatry, Habituation, Immersive simulation, Post-traumatic stress disorder, Predictive markers, Machine learning

## Abstract

Posttraumatic stress disorder (PTSD) is a complex mental health condition triggered by exposure to traumatic events that leads to physical health problems and socioeconomic impairments. Although the complex symptomatology of PTSD makes diagnosis difficult, early identification and intervention are crucial to mitigate the long-term effects of PTSD and provide appropriate treatment. In this study, we explored the potential for physiological habituation to stressful events to predict PTSD status. We used passive physiological data collected from 21 active-duty United States military personnel and veterans in an immersive virtual environment with high-stress combat-related conditions involving trigger events such as explosions or flashbangs. In our work, we proposed a quantitative measure of habituation to stressful events that can be quantitatively estimated through physiological data such as heart rate, galvanic skin response and eye blinking. Using a Gaussian process classifier, we prove that habituation to stressful events is a predictor of PTSD status, measured via the PTSD Checklist Military version (PCL-M). Our algorithm achieved an accuracy of 80.95% across our cohort. These findings suggest that passively collected physiological data may provide a noninvasive and objective method to identify individuals with PTSD. These physiological markers could improve both the detection and treatment of PTSD.

## Introduction

Posttraumatic stress disorder (PTSD) is a mental health condition that may occur in individuals exposed to traumatic events such as accidents, combat situations, or sexual abuse^1^. PTSD is a heterogeneous disorder^2–5^with symptoms including avoidance, hyperarousal or numbness and negative mood changes, which can lead to mental or physical health problems for affected individuals, as well as a socioeconomic burden on society^6^. According to recent studies, more than 6% of the United States (U.S.) civilians will develop PTSD in their lifetime^7,8^. Veterans are particularly prone to PTSD, with an estimated lifetime prevalence as high as 7% for male veterans, and 12% for female veterans^9^. PTSD can be difficult to diagnose because of its complex clinical manifestations and the reliance on primarily subjective assessment methods^10–12^. Moreover, the complex symptomatology of PTSD overlaps with that of other disorders, making establishing causal links difficult^13^. Furthermore, symptoms are often experienced outside clinical environments or therapeutic sessions, making accurate estimation difficult^14^. Objective tests, which are based mainly on blood or saliva samples are available^15,16^, but they are time-consuming and costly, making these methods less accessible and unsuitable for scaling to test a larger population. Consequently, there is an urgent need for objective and scalable methods to diagnose PTSD to better characterize, evaluate and treat patients.

Biomarkers can help identify different subtypes of PTSD, which can be crucial for understanding the variability in PTSD symptoms and severity. Many studies have attempted to enhance diagnostic capabilities and discover physiological markers to better characterize PTSD. Physiological data captured with sensors is central to stress-inducing protocols and constitute a promising path to understanding the link between the body’s response to stress and PTSD. A relationship between PTSD and both cardiovascular responses and changes in skin conductance have been demonstrated, such as in the work of^17^. Specifically, patients with PTSD reportedly experience increased resting heart rates (HRs), increased startle reactions, and increased HRs and blood pressure in response to traumatic stimuli^18–21^. Increased arousal and anxiety responses are more pronounced in individuals with PTSD during tasks involving the perpetual tracking and detection of threatening targets^22–25^. A recent study^26^investigating the behavioral and physiological responses to threatening virtual reality (VR) scenarios reported that the galvanic skin response (GSR), which conveys relevant information about the sympathetic nervous system activity^27^, was the most sensitive measure of the visual threat response. In comparison, the authors reported that the HRs and respiration rates of PTSD patients were more highly correlated with surprising events that occurred in a visual experimental environment. Since the GSR was the most threat-sensitive response, the authors used it as an output of physiological arousal in their study. Additionally, an increased GSR following exposure to a traumatic event was significantly correlated with the development of PTSD^28^. Furthermore, compared with those without PTSD, veterans with PTSD have been shown to display greater skin conductance reactivity while viewing combat-related VR scenarios than when viewing noncombat VR scenarios^29^, further suggesting heightened responsiveness toward trauma-specific cues. In addition to physiological changes in cardiovascular and skin conductance responses, eye tracking provides useful indicators of PTSD. PTSD patients maintain attention longer on negative stimuli^30^, and those with greater PTSD severity have increased pupil dilation when viewing images with negative valence and tend to view combat- or trauma-related images first^31^. It is also worth noticing that immersive simulations combined with physiological measurements have also been done in previous studies in the field of emotion recognition to induce stress or emotional reactivity^32–34^ with successful results, which reinforces the interest in using such paradigms.

Artificial intelligence (AI) and machine learning (ML) are powerful tools to investigate the importance of physiological markers with the promise of improving the accuracy of PTSD diagnosis and the prediction of onset and relapse. Within the field of ML, recent studies have reported promising results in the prediction of PTSD severity^35–38^. Schultebraucks *et al.*^37^ used ML on facial features, speech and natural language content during qualitative interviews to detect PTSD on patients in Trauma Unit with an accuracy of 83%. Li *et al.*^38^show promising results at predicting PTSD status with ML on firefighters using physiological signals such as EOG and ECG in a fear-inducing experiment with a ROC AUC score of 0.93. However, most efforts to predict PSTD severity with ML algorithms were not based on physiological markers. In an extensive review analyzing the use of ML for the diagnosis and evaluation of PTSD patients, 41 publications were included^36^. Among these studies, 21 used neuroimaging, 6 used clinical interviews, 8 used self-report questionnaires, and 6 used blood markers, facial features, social media data or electromagnetic resonance (EMR) to diagnose PTSD, with various success rates and prediction accuracies. Studies using neuroimaging or clinical interviews have reported promising results; however, these types of studies require expensive equipment or trained professionals and therefore are mostly based on small datasets. On the other hand, studies based on self-report questionnaires usually have larger sample sizes but lower accuracies. Inherently, subjectiveness in responses increases the risk of internal bias. While these studies reveal the promising potential of ML to predict PTSD severity, the applicability of ML in real-life ambulatory settings remains limited because of the time and cost required to prepare the data, preventing real-time or near real-time feedback. In another review listing the applications of ML for PTSD^35^, the authors analyzed 31 studies where ML algorithms were able to discriminate PTSD, with an overall accuracy between studies of 0.89 (95% confidence interval (CI) of [0.88, 0.91]). Most of the studies were also using neuroimaging data (17 over 31), but best accuracies were obtained using multi-dimensional data (using a mix of scales and EEG data), and none of the studies listed used physiological signals that can be readily captured by wearable devices (e.g., cardiovascular signals, changes in skin conductance).

To close these gaps, the combination of ML algorithms with passive monitoring of physiological signals to monitor PTSD symptoms could be a step forward in the discovery of objective diagnostic and predictive metrics of PTSD. Zheng *et al.*^39^ reported that nonintrusive devices, such as wearables, can improve the treatment of individuals and enable preemptive and predictive medicine. McWorther *et al.*^40^ proposed a monitoring system for detecting early signs of nightmares in individuals with PTSD. Although they did not attempt to detect PTSD, the authors proposed the application of ML optimization techniques on physiological signals (e.g., HR) monitored in real-time during sleep to take actions to wake patients when a nightmare was detected. Sadeghi* et al.*^41^, predicted hyperarousal events in veterans diagnosed with PTSD on the basis of HR and body acceleration signals captured by a wearable device via ML algorithms. Although their work confirmed the presence of PTSD markers in physiological signals, their model did not demonstrate the capacity to distinguish between healthy individuals and those with PTSD in their veteran populations.

On the basis of the abovementioned research results, we propose a novel ML algorithm that can predict the likelihood of PTSD symptoms from physiological signals such as cardiovascular responses, skin conductance and pupil diameter. Our ML design is based on the concepts of habituation and sensitization, which are central to the general understanding of trauma responses in clinical practice. In the neurobiological literature, habituation refers to the nonassociative learning process in which the magnitude of a response to a stimulus decreases with repeated exposure to the stimulus in comparison to the large response elicited by acute exposure^42,43^. The opposite phenomenon is called sensitization, i.e., an increased response to repeated exposure to a stressor.

Our algorithm also offers an explanation of the model’s prediction via explainable AI (XAI). Post hoc XAI provides an explanation alongside the ML algorithm in a way that is understandable by experts. Our model specifies the relationships between physiological features such as HR or the GSR and PTSD status and paves the way for future studies in ambulatory settings.

## Methods

### Study design

The primary aim of this study was to investigate how individuals with U.S. military experience who have PTSD differ from those who do not have PTSD in terms of visual processing and working memory. Participants completed a militarily-related visual search task that manipulated stress levels within an immersive desktop environment. The experimental conditions manipulated the stimulus context (neutral versus combat related) and stress level (low versus high). Several physiological measurements were taken throughout the experiment, including the three signals analyzed in this work: ECG, GSR and eye tracking.

### Participants

This study and all methods were carried out in accordance with the accredited Institutional Review Board at the U.S. Army Combat Capabilities Development Command Army Research Laboratory (ARL) and conducted in compliance with the ARL Human Research Protection Program and the Declaration of Helsinki. The protocol was approved by the US Army Research Laboratory Human Research Protection Program (ARL 21–104). Data were collected from 41 active-duty U.S. service members and veterans recruited from Joint Base San Antonio, Texas. Informed consent was obtained from all participants and all participants signed an Institutional Review Board approved consent form prior to participation. Consent forms and all experimental procedures were reviewed and approved by the U.S. Army Combat Capabilities Development Command (DEVCOM) Army Research Laboratory (ARL) Human Research Protections Program (ARL 21–104). Participants were classified as either having PTSD or not having PTSD on the basis of a threshold set to 36^44^applied to the score obtained through their self-reported responses to the PTSD Checklist for Military version (PLC-M)^45^. The demographic and clinical characteristics of the participants are detailed in Table 1. Among the 41 participants included, 4 did not complete the PCL-M, 12 had one or several physiological signals that were incorrectly measured (either a technical problem during the experiment or a data file that was corrupted during saving), and 4 had no events recorded during the simulation. The 21 remaining participants were considered in our results and analysis. The majority of the cohort was male (90.48%), the average age was 51.43 ± 13.92 years, and the median age was 54 (range: 26–69) years. The average PCL-M score was 32.48 ± 13.14, with a median score of 29 (range: 17–54) and after application of the cut-off, 10 (47.6%) participants were considered having a PTSD. Among this population, 61.9% were veterans and 57.14% had previous combat experience.

**Table 1:** Baseline demographics and clinical characteristics.

**Variables**	
Mean (±SD) age, years	51.43 ± 13.92
Median (min–max) age, years	54 (26–69)
Mean (±SD) PCL-M score	32.48 ± 13.14
Median (min–max) PCL-M score	29 (17–54)
PTSD, yes:no, n(%)	10 (47.6%) : 11 (52.4%)
Combat experience, yes:no, n (%)	12 (57.14%) : 9 (42.86%)
Mild Traumatic Brain Injury, yes:no (%)	3 (14.29%) : 18 (85.71%)
Mean (±SD) deployments, number	2.14 ± 2.3
Median (min–max) deployments, number	2 (0–10)
Sex, M:F, n (%)	19 (90.48%) : 2 (9.52%)
Mean (±SD) time in service, years	18.24 ± 9.69
Median (min–max) time in service, years	20 (1–36)
is a veteran, yes:no, n (%)	13 (61.9%) : 8 (38.1%)
is an officer, yes:no:missing, n (%)	2 (9.52%) : 16 (76.19%) : 3 (14.29%)

### Study procedure

Each participant engaged in the visual search task for approximately 30 minutes. During the task, the participants completed four conditions in which the stimulus context and stress level were manipulated. The participants completed either the neutral or combat levels first (counterbalanced). High-stress conditions always preceded low-stress conditions. For example, a participant assigned to complete the neutral conditions first would complete the high-stress neutral condition, the low-stress neutral condition, the high-stress combat related condition, and finally the low-stress combat related condition. The participants were tasked with finding an assigned target vehicle within each condition. In the high-stress conditions, the participants were exposed to high-stress trigger events involving explosions, firing tanks, or barking dogs.

Several physiological signals were captured throughout the experiment. In this work, we focused on signals measured by eye tracking, electrocardiogram (ECG), and GSR sensors. Eye tracking was performed via the Tobii Pro Spectrum. ECG and GSR data were recorded via the Biosemi Active two System. Because the participants needed to use their fingers to complete the tasks (e.g., to use computer keys and a mouse to navigate around the virtual environment), the GSR was collected by placing two sensors on the thenar and hypothenar eminences of the left hand. ECGs were collected through a single electrode placed at the V4 location on the chest, one of the standard ECG chest electrode positions^46^. Following their participation, active-duty participants were eligible to receive a Certificate of Achievement, whereas veterans were monetarily compensated for their participation.

### Physiological data and events

**Fig. 1:**
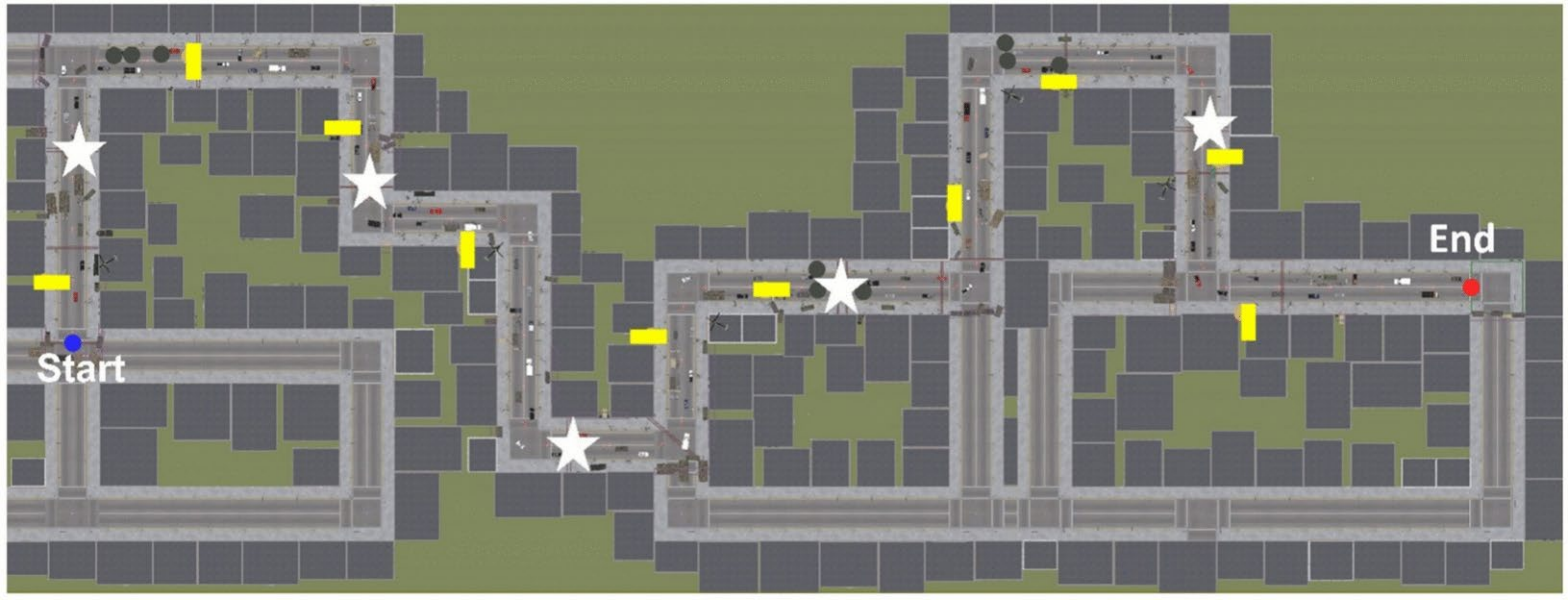
Top-down view of the virtual immersive simulation. The participants were presented with this novel and real-world complex environment on a desktop screen. Their task was to search a scene for specific objects (e.g., all motorcycles or cars) while they were navigating the environment. The pacing of the search task was left to the participant, as was the navigation of the environment. Navigation cues (e.g., trail blazes) were provided to assist the participants and to provide a common perceptual target across all participants. Navigation was performed via standard game control keys (i.e., the W, A, S, and D keys), and the mouse was used to change the view or turn around. When the participant navigated near a navigation cue, a direction mark was displayed on the cue to indicate which specific direction the participant should be heading.

The analyses presented in this paper specifically focused on the high stress combat condition. As stated previously, this condition involved stress-inducing military stimuli and events. An event was defined as a moment recorded by the simulation software when a particular action occurred, for instance, the occurrence of a stress-inducing stimulus (e.g., a flashbang) or the participant leaving a specific zone in the simulation. The average time for all participants to complete this condition was 7.86 ± 1.83 minutes. The complete list of events that occurred during this condition is available in Table 1 in the Supplementary Material, and a top-down view of the virtual environment is presented in Fig. 1. The decision to focus on this scenario was motivated by the theory that trauma-related cues may elicit stronger stress responses; therefore, in the military population, we anticipate that this condition has the greatest potential to elicit a stress response. Additionally, focusing on the high-stress military-related condition allows us to reduce unnecessary noise (e.g., events not a priori associated with stress) within the data that, if included, would increase the difficulty of the ML model task. Moreover, including other conditions than high-stress military-related would have increased the number of possible missing data, resulting in the possible complete removal of the participants impacted with too many missing data from the data to train the model if no imputation was possible. Additionally, we chose to remove all events corresponding to withdrawal from a triggering zone since these events only involved information provided by the simulation software and thus should not elicit a physiological response.

For each participant, ECG and GSR signals were delivered at 2048 Hz, and eye tracking was performed at 300 Hz. Meaningful physiological time-series were obtained from these signals in the following manner:

- HR data were extracted from ECGs via standard processing methods (e.g., peak detection and correction).
- Phasic skin conductance measurements, which are generally associated with short-term events, were computed via the convex optimization package cvxEDA^27^. Phasic GSR refers to the rapid, transient changes in skin conductance that occur in response to stimuli or specific events; these changes reflect autonomic nervous system activity and are typically associated with short-term arousal or stress responses.
- Pupil diameter data were extracted from the eye tracking sensor data.

**Fig. 2:**
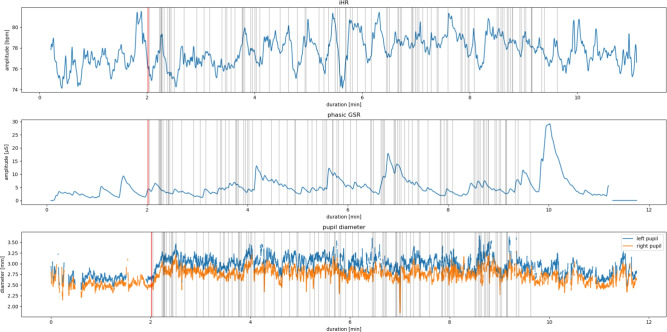
Examples of the physiological signals used in this work. The HR was computed from an ECG signal captured at 2048 Hz. The phasic GSR was obtained from the raw GSR data collected at 2048 Hz via standard decomposition techniques (i.e., the convex optimization approach^27^). The pupil diameter was captured with the eye tracking sensor at 300 Hz. All three signals were downsampled at 32 Hz. The gray vertical lines correspond to the occurrence of an event during the simulation, and the red line corresponds to the event ‘GAMESTART’, i.e., at the beginning of the simulation.

These three signals were downsampled at 32 Hz before further processing. An example of these signals is shown in Figure 2. We refer to HR, phasic GSR and pupil diameter in this work as our raw physiological data.

### Preprocessing

**Fig. 3:**
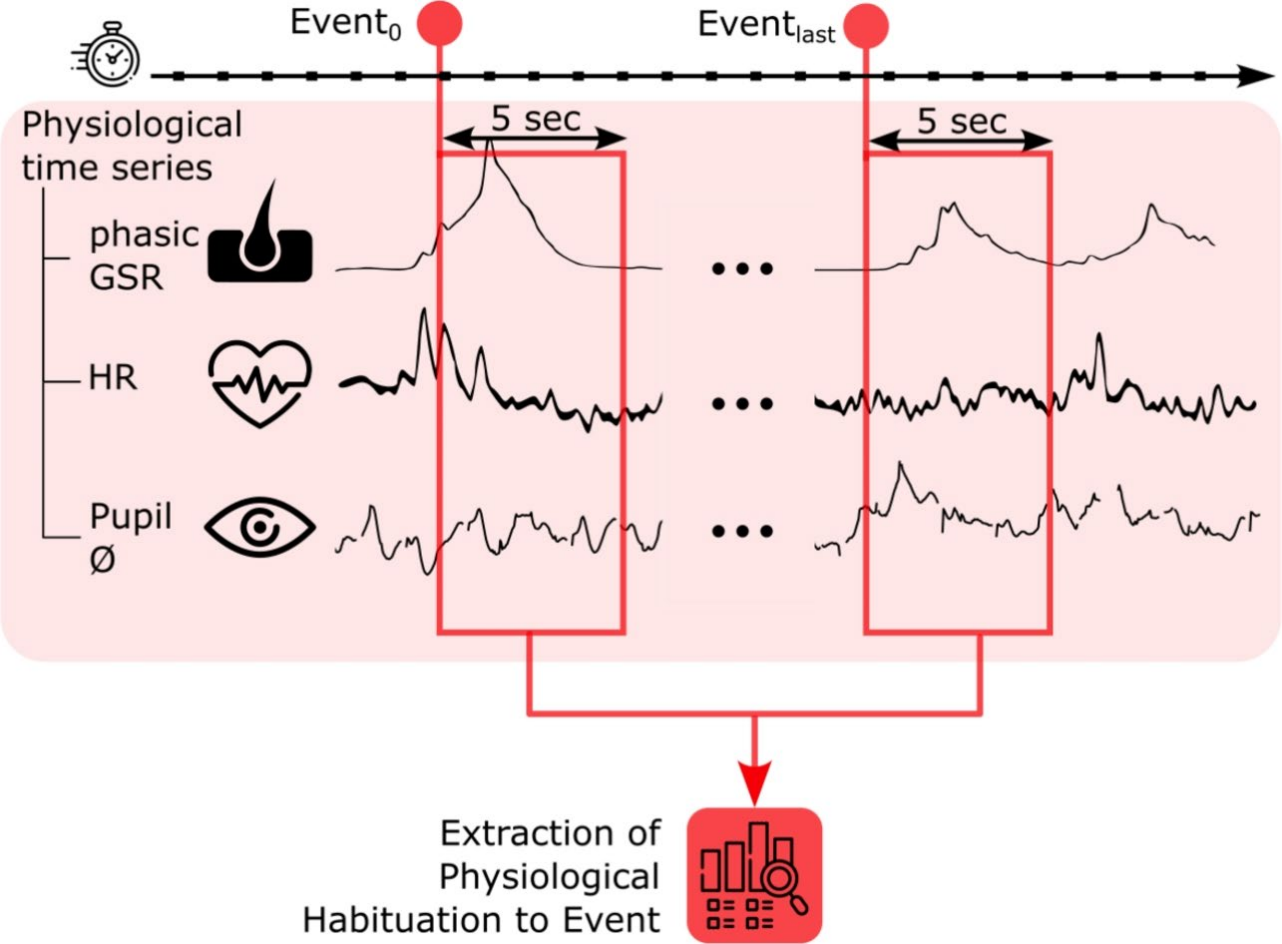
Schematic of how the habituation feature was computed from the physiological time series HR, phasic GSR, pupil diameter and event occurrence. $\mathrm {Event_0}$ and $\mathrm {Event_{\textrm{last}}}$ correspond to the first and last events of a given type, respectively, that occurred during the simulation.

After the raw signals were obtained from the physiological sensors under the high-stress combat-related condition, multiple preprocessing steps were performed before our ML model was applied. We then computed the physiological habituation from HR, phasic GSR, and pupil diameter. We defined the physiological habituation features as the difference in the physiological response in HR, GSR and pupil diameter values five seconds after two events of the same nature that occurred at different times during the simulation (e.g., ‘explosion’ and ‘flashbang’). A positive value corresponded to habituation to the event, whereas a negative value corresponded to sensitization (i.e., negative habituation) to the event. A duration of five seconds was chosen to ensure that the reaction to the stimulation was captured entirely, especially for the GSR signal, which can involve a slow reaction time usually ranging from 1 to 5 seconds^47^. An illustration of how habituation was computed on the signal is available in Fig. 3. Next, we extracted meaningful features from these raw signals, namely:

- the mean/max/min HR and phasic GSR during the five-second window after an event.
- the number of missing data points in the pupil diameter during the five-second window after an event, which was a proxy for the eye blinking rate.

For example, the features of habituation to a given type of event stimulus (e.g., ‘flashbang’) was defined as:

$$\begin{aligned}&\textrm{max}(\textrm{phasic}[t_0:t_0+5 \ \textrm{sec}]) - \textrm{max}(\textrm{phasic}[t_{\textrm{last}}:t_{\textrm{last}}+5 \ \textrm{sec}]) \\&\textrm{min}(\textrm{phasic}[t_0:t_0+5 \ \textrm{sec}]) - \textrm{min}(\textrm{phasic}[t_{\textrm{last}}:t_{\textrm{last}}+5 \ \textrm{sec}])\\&\textrm{mean}(\textrm{phasic}[t_0:t_0+5 \ \textrm{sec}]) - \textrm{mean}(\textrm{phasic}[t_{\textrm{last}}:t_{\textrm{last}}+5 \ \textrm{sec}])\\&\textrm{max}(\textrm{HR}[t_0:t_0+5 \ \textrm{sec}]) - \textrm{max}(\textrm{HR}[t_{\textrm{last}}:t_{\textrm{last}}+5 \ \textrm{sec}]) \\&\textrm{min}(\textrm{HR}[t_0:t_0+5 \ \textrm{sec}]) - \textrm{min}(\textrm{HR}[t_{\textrm{last}}:t_{\textrm{last}}+5 \ \textrm{sec}])\\&\textrm{mean}(\textrm{HR}[t_0:t_0+5 \ \textrm{sec}]) - \textrm{mean}(\textrm{HR}[t_{\textrm{last}}:t_{\textrm{last}}+5 \ \textrm{sec}])\\&\textrm{EBP}_{t_0} - \ \textrm{EBP}_{t_{\textrm{last}}} \end{aligned}$$

where ‘phasic’ stands for phasic GSR, ‘EBP’ stands for ‘eye blinking proxy’, and $t_0$ and $t_{\textrm{last}}$ are the times of occurrence of the first and last event types considered in the simulation, respectively.

Following these definitions, the input data given to the ML pipeline were then defined as pairs of event type/physiological markers (e.g., explosion/max HR or flashbang/mean phasic). In the remainder of this work, importantly, a feature (i.e., an event/physiological marker pair), was defined as *”physiological habituation to a given event expressed by the physiological marker of the participant”*.

Once the physiological habituation features were computed, a leave-one-out (LOO) scheme was adopted for the remainder of the pipeline, meaning that when a participant was included in the test set, the model was trained on all other participants except that participant. This is a standard procedure to ensure that data leakage does not bias the results. The final metric considered was the average accuracy on the test set over all LOO runs. The labels given to the ML model were the Boolean values representing the participant’s PTSD status. The PCL-M total score ranged from 17–85^45^, and a participant was considered to have a PTSD when their PCL-M score was greater than 36^44^. Therefore, we defined our Boolean label as follows:6

1 $$\begin{aligned} {\left\{ \begin{array}{ll} & y= 1 \ \text { if PCL-M score }> { 36}\\ & y= 0 \ \text { if PCL-M score } \le 36 \end{array}\right. } \end{aligned}$$

A statistical t-test with p-value threshold of $\alpha = 0.05$^48,49^ was then performed to remove the features that were not statistically significant with respect to the training labels. This step was necessary to not overwhelm the model with meaningless training features since it is reasonable to hypothesize that not all event types triggered a reaction (see Table 1 in the Supplementary Material for a complete list of events), as well as to counter the possible curse of dimensionality because the number of feature dimension was greater than the number of samples. Finally, standard normalization was performed before training the ML model.

### Machine learning classifiers

Several ML classifiers were trained to predict PTSD status, including K-nearest neighbor (KNN), random forest, decision tree, logistic regression, multilayer perceptron and Gaussian process classifiers, as well as a combination of these classifiers via soft or hard voting procedures. For further details regarding these standard ML models, please refer to the book by Geron A^50^.. The hyperparameters were optimized via the HyperOpt python library^51^. Overall, the best results were obtained with the Gaussian process classifier, a nonparametric, probabilistic model that uses a joint Gaussian probability distribution to define a prior over function, which was further updated with observed data to make predictions about class membership probabilities.

### Model interpretation

Since the Gaussian process classifier is akin to a black box machine, once the model was trained and the left-out test participant was evaluated, it was necessary to understand the predicted result and to obtain an explanation of the features’ influence on the model predictions. We used SHapley Additive exPlanations (SHAP) to address this need. SHAP uses game theory concepts to allocate values to features in a model on the basis of their importance in prediction. Please refer to the original reference for details about SHAP^52^. SHAP values indicate how much each feature contributes to the prediction of an ML algorithm (i.e. its importance in the prediction process of the model). The complete pipeline from the raw physiological time series to the final prediction result is illustrated in Fig.1 of the Supplementary Material.

## Results

### Participant database

For each participant, the database included the simulation time for the high-stress condition, physiological data collected during that time, the PCL-M score and the PTSD status (PTSD or non-PTSD). Specifically, the database was composed of 7.86 ± 1.83 minutes of physiological data collected during the high-stress combat-related condition, with a median duration of 7.35 (range: 6.09–14.10) minutes and a PCL-M score reduced to a Boolean label (as described in Equation 1). After performing the processing and feature selection steps, the average number of remaining features per participant was 16.95 ± 2.32, with a median of 18 (range: 12–21).

### Model performance

**Fig. 4:**
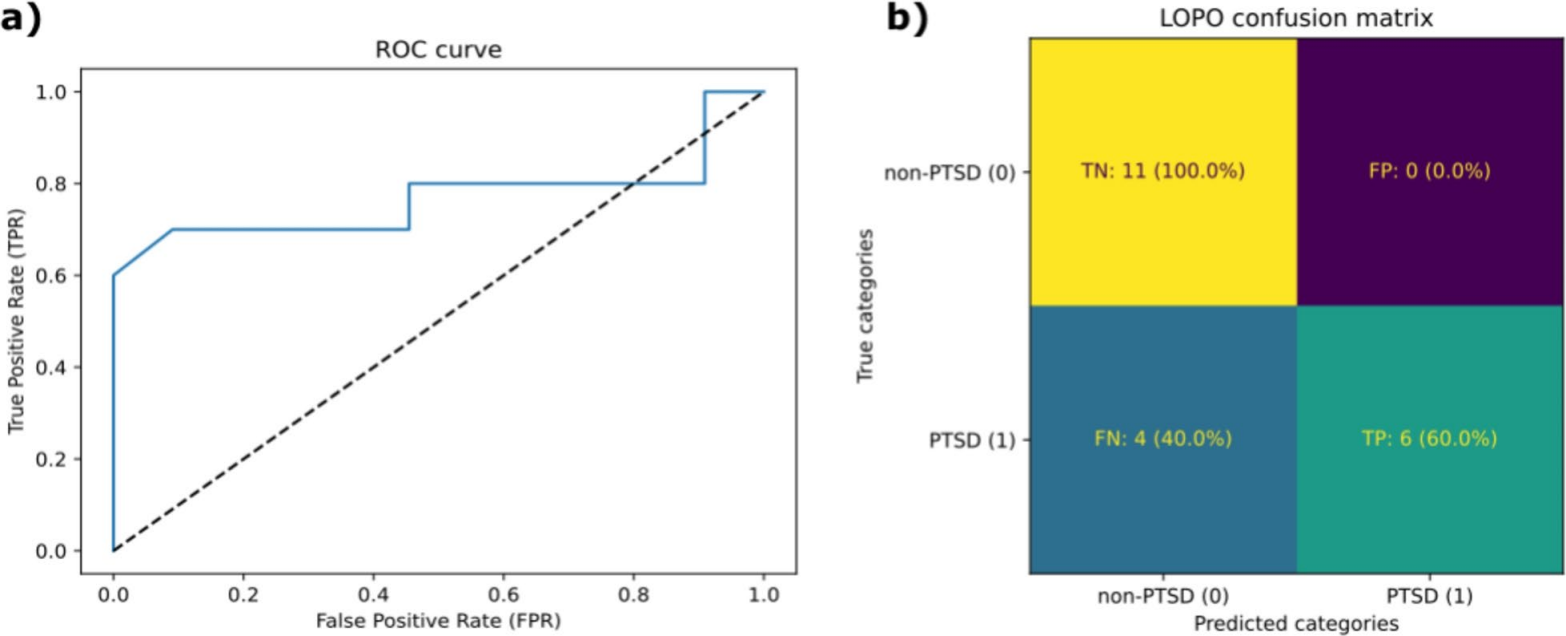
**(a)** Left: ROC curve of the prediction model. **(b)** Right: Confusion matrix (with normalized values in parentheses) of the model’s binary prediction for the test participants. With a binary accuracy of 80.95%, the model had a true negative rate (TNR) of 100% but a true positive rate (TPR) of 60%, meaning that 4 out of 10 participants with PTSD were not well detected. However, the ROC curve on the left indicates that this value could be increased to 80% at the cost of a slightly deteriorated false positive rate, meaning that more participants without PTSD would be predicted to have a PTSD. Depending on how important not missing any potential participants with PTSD is considered in the model, possibly optimal models could be fine-tuned with the desired TPR/TNR thresholds.

The optimal ML model selected among the ML classifiers tested (see list in section **Machine learning classifiers**) was a Gaussian process classifier model. The model achieved an overall binary accuracy of 80.95%, whereas the accuracy of the baseline constant prediction of the majority class was 52.4%. The normalized confusion matrix of the binary prediction (PTSD/no-PTSD) is shown on the right side of Fig. 4. A perfect prediction corresponds to a diagonal matrix with ones over the diagonal. The model had a true positive rate (TPR) of 60% and a true negative rate (TNR) of 100%. The receiver operating characteristic (ROC) curve of the model is shown on the left side of Fig. 4, with a diagonal dashed line corresponding to a random prediction.

### Prediction explanations

**Fig. 5:**
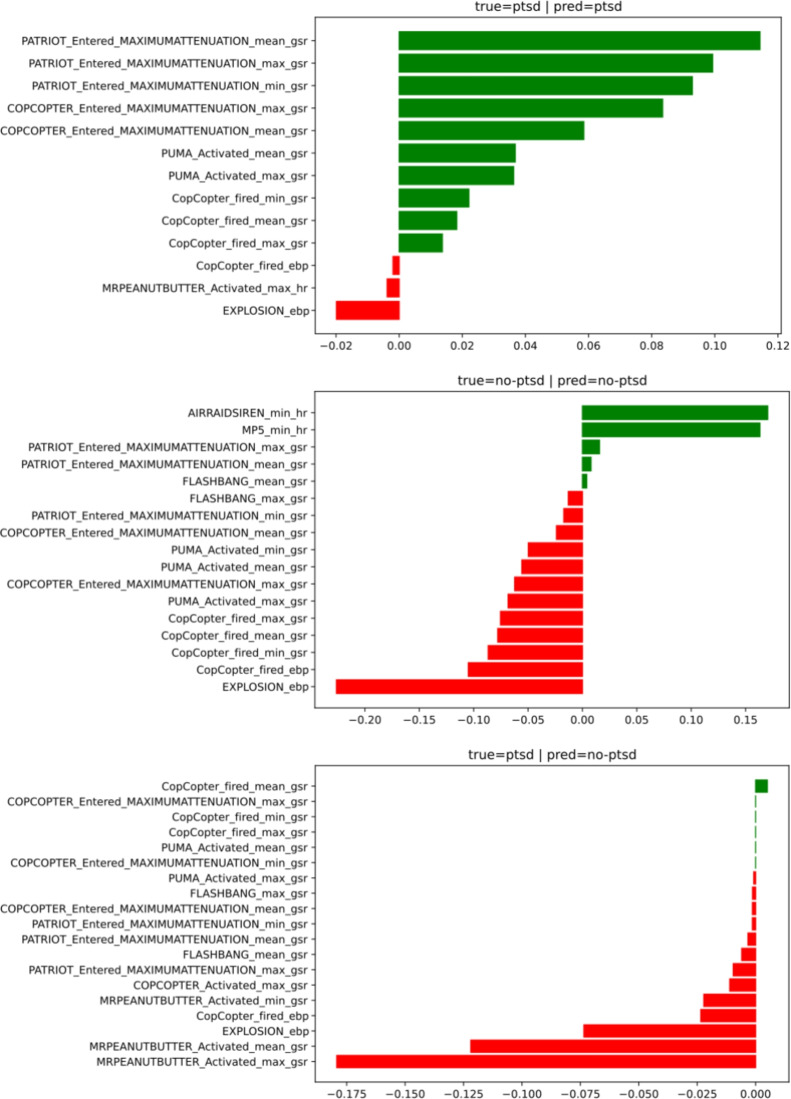
SHAP explainability algorithm outputs for 3 different test participants. The example on top is for a participant who declared having a PTSD correctly predicted by the ML model. The middle example is for a participant without PTSD, which was also correctly predicted. Finally, the last example is for a participant who declared having a PTSD incorrectly detected as not having PTSD. The x-axis corresponds to the importance value determined via SHAP to the features on the y-axis. If the feature’s value is positive (in green), it supports the prediction of class 1 (PTSD), and if the feature’s value is negative (in red), it supports the prediction of class 0 (non-PTSD). The higher the absolute value of the feature is, the more important it is considered by the model to make its prediction. In these examples, the model’s prediction is always supported by several features, and their importance varies across the participants, which could be interpreted as an individual stress signature.

The SHAP algorithm was used for Gaussian process classifier prediction for the left-out test participant. This explanation technique gives a list of features and their importance regarding model prediction. Several examples of these explanations are presented in Fig. 5, illustrating different prediction cases (a true positive, a true negative and a false negative). Positive value features are features that support the model to predict class 1 (PTSD), whereas negative value features orient the model to predict class 0 (non-PTSD), and the higher the absolute value is, the more important the feature is in the model.

**Fig. 6:**
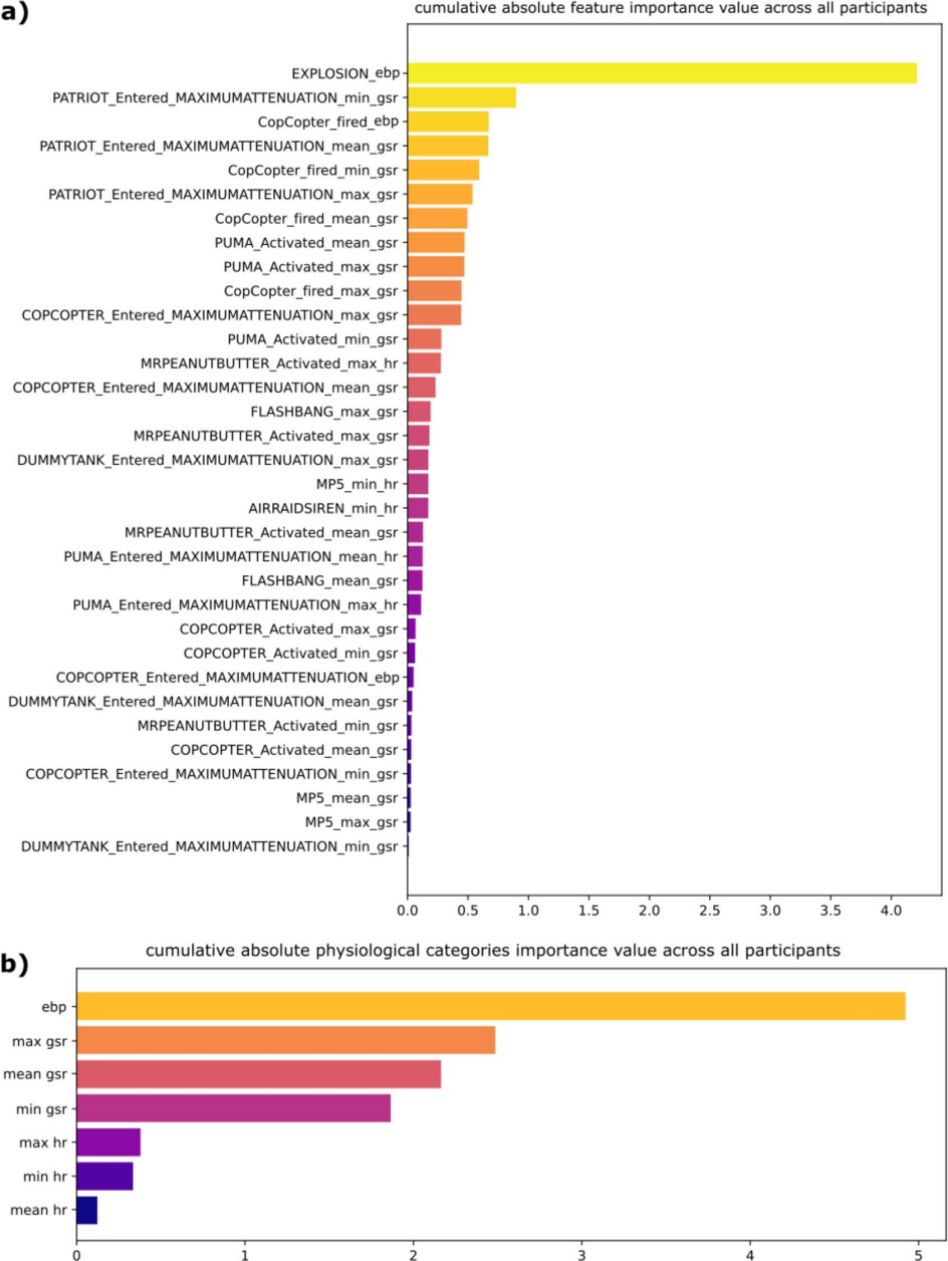
**(a)** Top: Ranking of the cumulative absolute importance values of the features during the whole simulation for all participants. For each feature composed of an event/physiological marker pair, we summed the absolute importance value determined via SHAP for each test participant. The results provide an overview of the most important features for predicting PTSD or non-PTSD status across all participants and, therefore, which events are the most stressful, as well as which physiological features are the most related to these stressful stimuli. **(b)** Bottom: Ranking of the cumulative absolute importance values of the physiological features during the whole simulation for all participants. This result was obtained by grouping features of the same physiological category (e.g., max GSR, EBP, etc.). It provides an overview of which physiological features are the most predictive of PTSD or non-PTSD status across all participants.

Using these results, we also obtained a global importance ranking of the features during the simulation for all participants, i.e., which pairs of events/physiological markers (see complete list in Table 1 in the Supplementary Material) were the most predictive among all the participants. This result is shown in the top of Fig. 6. A similar result obtained by regrouping the features by the type of physiological markers (e.g., mean GSR, max HR, etc.) is illustrated on the bottom of Fig. 6.

### Individual habituation response

**Fig. 7:**
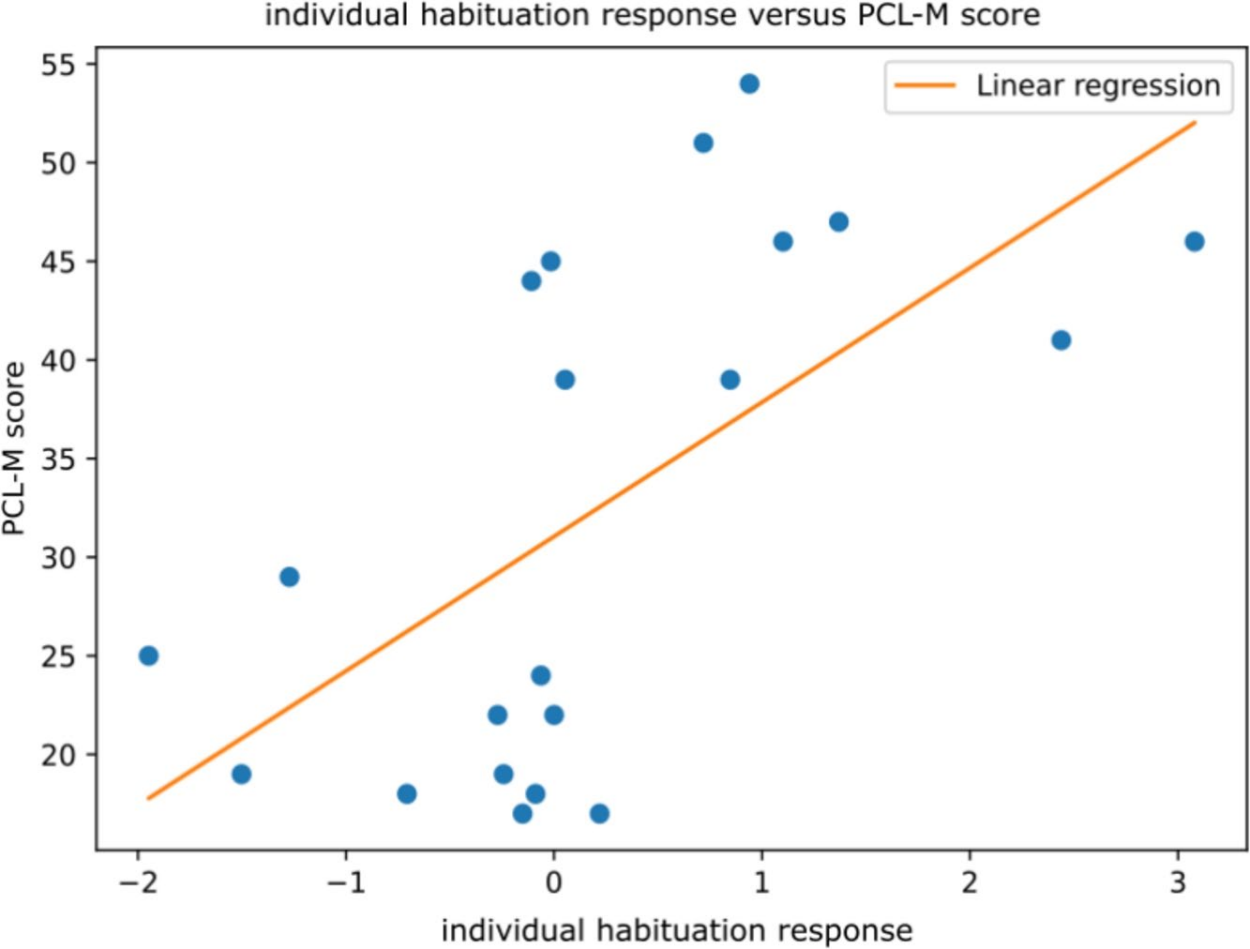
Individual habituation response as defined in Equation 2 versus the reported PCL-M score. A negative value on the x-axis means that the participant tends to have overall sensitization (i.e., negative habituation) to stress-inducing events during the scenario, whereas a positive value indicates a tendency to habituation to stress-inducing events. The orange line represents the linear regression of the scattered points in blue. These findings indicate that participants with low PCL-M scores tend to be more sensitive to stress stimulation. A participant was considered to have a PTSD when the PCL-M score was greater than 36.

Finally, still using prediction explanations from the SHAP algorithm, an individual habituation response was calculated for each participant by aggregating the feature values weighted with the absolute value of the corresponding SHAP output:

2 $$\begin{aligned} \textrm{individual \ habituation \ response} = \sum _i x_i\cdot |w_i| \end{aligned}$$

where $x_i$ is the habituation feature value of the pair event/physiological marker *i* (as detailed in the **Preprocessing** section) of the participant considered and $w_i$ is the corresponding importance value given to this feature by SHAP. The choice to weight the feature value by the absolute value of its importance determined via SHAP was made to favor features that had a meaningful impact on the model prediction, since it means that these features were considered by the model as triggering more physiological reactions to the participant than the other features. A comparison of the individual habituation response obtained with the PCL-M score for all participants was also performed. The results are displayed in Fig. 7.

## Discussion

In this work, we developed, evaluated and explained an ML algorithm to efficiently predict PTSD status among U.S. veterans and active-duty service members. We used physiological data passively collected during a controlled experiment where participants were immersed in a virtual, stress-inducing desktop simulation. The ground truth for PTSD status was self-reported according to the participants’ responses to the PCL-M^44^. After preprocessing the raw physiological signals, we calculated the physiological habituation to the different types of events that occurred during the simulation. We then performed a selection on the resulting features, and we trained our ML model via an LOO scheme, ensuring results that are robust and free from data leakage. Our Gaussian process classifier yielded an accuracy of 80.95%, with a TPR of 60% and a TNR of 100% (confusion matrix and ROC curve in Fig. 4). Post hoc SHAP also allowed us to sort the most important stimulating events during the simulation for all participants, as well as the most important physiological signals for the prediction of each participant’s PTSD status.

Our results on the participants’ individual habituation responses during the whole simulation, as computed via Equation 2indicated that none of the participants predicted as having a PTSD by the model experienced negative habituation (i.e., sensitization), whereas 73.3% of the participants predicted as not having a PTSD experienced sensitization to stressful events. When considering instead the true categories PTSD/non-PTSD obtained via the self-reported PCL-M, 20% of the participants having PTSD and 81.2% of the participants without PTSD experienced sensitization to stressful events during the experiment. The difference observed between the model predictions and participants reports (i.e., the ground truth label reported via the PCL-M) can be explained by both the known bias of self-administered questionnaires^12^ and the incertitude of the model. Moreover, further analysis indicated that there was a relationship between the intensity of the individual habituation response and the PCL-M score (see Fig. 5). A greater individual habituation response (positive habituation to stress-inducing events during the simulation) was correlated with a higher PCL-M score (i.e., increased symptoms of PTSD), whereas a lower habituation response (i.e., sensitization to stress-inducing during the simulation) was correlated with a lower PCL-M score. Considering that all the participants were either active military or former military personnel, the difference in habituation to stressful events during the scenario cannot be attributed to the lack of military training. Therefore, this result is likely linked to PTSD severity. Previous studies have demonstrated the link between PTSD symptoms severity and habituation to stressful stimuli, however both negative and positive association have been observed. Authors in^43,53,54^observed a positive association between habituation and the slope of PTSD symptoms while a negative association was demonstrated in other studies^55–57^. The link between the amygdala and habituation to stimuli has also been demonstrated before^58^, and PTSD has been reportedly associated with the inhibition of the transmission of stress-related information to the amygdala^59,60^. Therefore, the diversity of results observed in the literature and in our study may come from various factors such as the heterogeneity of PTSD symptoms’ expression, the cohort (with different trauma-related experiences and/or different populations) or the protocol itself.

The results obtained validated the existence of a link between habituation and PTSD status. Moreover, the individual habituation response we introduced in Equation 2 was revealed to be directly related to PTSD severity, confirming results from the literature establishing a relationship between habituation intensity and PTSD severity. Finally, our work showed that a participant’s habituation process is a mechanism that is observable in readily measurable physiological signals such as the HR and GSR, which require less cumbersome equipment than that usually used for neurological signals (EEG, functional magnetic resonance imaging, etc.) to measure, for instance, the reactions of the amygdala. This work paves the way for the use of dedicated wearable medical devices in conjunction with ML algorithms to follow-up patients experiencing PTSD or to assess the progression of the disorder.

An analysis of the SHAP results indicated that the feature ‘explosion/EBP’ was the most predictive of PTSD status, followed by the GSR features. A detailed analysis of this result is presented in the Supplementary Material and revealed that the event labeled ‘explosion’ was the event that induced the most stress for the participants and therefore was the most predictive of PTSD status during this particular virtual simulation. However, the feature selection process also revealed that this event was the most significantly related to PTSD status when associated with eye blinks. The link between auditory stimuli and eye blinking has been extensively studied in the literature, with eye blinking marking a period of cognitive processing following stimulus occurrence^61–64^. Thus, this result could indicate that the ‘explosion’ event imposed a particular cognitive load on the participants. Further testing of the model after excluding this particular event from the data demonstrated that the GSR was, in this case, the most important physiological feature, which is consistent with prior studies^26,28,29^, considering that GSR signals are able to capture stress conditions.

Our work has several limitations. The cohort of 21 analyzed participants is small, even if the LOO procedure ensures good generalizability of the method to new participants. Moreover, while we used a military population and military stimuli, there can also be variance in the results since PTSD patients can have different traumatic experiences activated with specific trauma-related stimuli and different physiological responses since PTSD is a heterogeneous disorder^2–5^. The results obtained, and particularly the relation between PTSD severity and habituation, could therefore be refined by including more participants, if possibly from different study sites. To overcome the heterogeneity, we could also study the reaction to more individualized trauma-related stimuli, or to the opposite, look for reactions that do not depend on specific trauma, such as acoustic startle reflex^34^.

The sex distribution was far from balanced (see Table 1), whereas studies estimate that lifetime PTSD prevalence in women could be 6% higher than that in men^65^. Concerning the protocol for the experiment itself, the simulation time was short, with a mean duration of 7.86 ± 1.83 minutes. Consequently, we could not use heart rate variability (HRV) features since they are commonly computed across at least five minutes of cardiac signals, and the lower importance of cardiac features in our results is probably linked to this fact, whereas many studies have shown that HRV is strongly related to stress and PTSD status^18–20,66^. The ML model achieved good predictive performance with an accuracy of 80.95%, but had a low TPR of 60%, which increased to 80% with a correct threshold for the TPR/FPR, as presented in the ROC curve in Fig. 4. Additionally, the labels used were based on the self-reported PCL-M, and participants tend to score higher on this scale than on the Clinician-Administered PTSD Scale for the DSM-5 (CAPS-5)^12^, possibly leading to participants without PTSD being classified as having PTSD and consequently making the model task more difficult, as well as introducing an incertitude in the results obtained on the relationship between habituation and PTSD severity.

In conclusion, in this work, we confirmed that physiological habituation is a marker of PTSD status, validating our original definition of habituation compared with the existing PTSD literature studying this phenomenon (HR, phasic GSR and eye tracking versus cortisol levels). Using only passively collected data, we also paved the way for the development of a system that can predict the progression of PTSD and PTSD severity on the basis of physiological data captured by a wearable monitoring device. Moreover, when considering the ability of a model to predict the PTSD status of outpatients via physiological markers, an analysis of feature importance for PTSD expression, as presented in this work, may allow for better personalized follow-up and treatment. With respect to clinical use in the field, this system could be further enhanced by removing the need for labeled events for the model to make its prediction. The automatic detection of hyperarousal events in an ambulatory, free-living setting has been explored in other studies and could lead to better integration of our work into clinical practice.

## Supplementary Information

Supplementary Information.

## Data Availability

The data cannot be shared publicly because of US Army Research Laboratory Human Research Protection Program IRB restrictions. The data for this study are stored in a secured laboratory facility in physical form, as well as in an online secured data repository. Data sharing agreements may be put in place for researchers who meet the criteria for access to confidential data. Interested parties may contact Heather Roy (heather.e.roy2.civ@army.mil).
