# Supplementary material for: Assessment of PTSD in military personnel via machine learning based on physiological habituation in a virtual immersive environment
Source: Sci Rep. 2025 Mar 4;15:7562. doi: 10.1038/s41598-025-91916-x (PMC11880569; doi:10.1038/s41598-025-91916-x)
Supplement: Supplementary file 1 — Supplementary Information. [file 41598_2025_91916_MOESM1_ESM.pdf]

Assessment of PTSD in Military Personnel via Machine Learning Based on Physiological Habituation in a Virtual Immersive Environment

Gauthier PELLEGRIN[1,*], Nicolas RICKA[1], Denis A. FOMPEYRINE[1], Thomas ROHALY[2], Leah ENDERS[3], and Heather ROY[3]

[1]MyndBlue, F-75008 Paris, France
[2]DCS Corporation, Alexandria, Virginia, United States of America
[3]Human in Complex Systems Division, DEVCOM Army Research Laboratory, Aberdeen Proving Ground, Maryland, United States of America
[*]Corresponding author: gauthier@myndblue.ai

Supplementary material

Feature analysis

With respect to the results obtained with the standard pipeline in this work, the feature formed by the pair 'explosion' event and eye blinking proxy (EBP) was a strong predictor of PTSD status, at least in the setup of this experiment. Therefore, several questions have arisen regarding this information:

- Why is that feature so predictive of PTSD condition in this experiment, and what is the link between the 'explosion' event and EBP, since the event involved a sound stimulus and not a visual stimulus (as opposite to the 'flashbang' event for instance)?
- Does the predictive power of this feature lie in the event, the physiological marker, or both?
- What is the predictive power of the remaining features?

To answer these questions and better understand the importance of this event/physiological marker pair in this setup, we performed several tests on the pipeline described in this work.
First, we decided to keep only the 'explosion' event in our pipeline. The resulting binary accuracy of this model was 66.67%. Interestingly, the possible features with this event should have been the following pairs:

- explosion/max heart rate (HR), explosion/mean HR, explosion/min HR
- explosion/max phasic galvanic skin response (GSR), explosion/mean phasic GSR, explosion/min phasic GSR
- explosion/EBP

However it appeared that only the habituation feature explosion/EBP successfully passed the statistical t-test during the feature selection process (i.e., only one feature remained, and the other features with HR and phasic GSR were dropped during feature selection). Therefore, only the EBP physiological feature seemed linked to the 'explosion' event for the prediction of PTSD status.

The second test was performed to keep all events but remove EBP from the physiological features. Under these conditions, the accuracy of the model decreased to 33.3%. However, confirming the result of the previous test, it appeared that no 'explosion' event remained after the feature selection process. To allow more features to pass this selection, we tried a similar test but with a more permissive threshold on the statistical t-test(set to $\alpha = 0.1$ instead of 0.05). In that case, the accuracy rose to 47.62%, which is around the majority class prediction, and there were 'explosion' events in the features kept by the selection process. This result indicated that this event seemed more important than EBP itself for the model to predict PTSD status.

Finally, we removed 'explosion' from the list of events in the simulation. With this setup, the predictive accuracy of the model decreased to 38.10%, which was below random. This result seemed to confirm that, for this experiment, the physiological response to the 'explosion' event was indeed the strongest predictor of PTSD status.

To conclude, in this analysis of the features' predictive power, the most important feature in the prediction of PTSD status by the model was the 'explosion/EBP' feature, and more specifically, the 'explosion' event seemed to be very stress-inducing for the participants. The link between auditory stimuli and eye blinking has been extensively studied in the literature, and the

consensus is that it is a marker of a period of cognitive processing of the stimuli that has occurred [1–4]. This signifies that the 'explosion' was exercising a particular cognitive load on the participants compared with other events during the scenario. Finally, we can also conclude that physiological responses to other events in the scenario were also markers of PTSD status since the overall accuracy was 80.95%, i.e. more than 14% above the result with 'explosion' only.

To illustrate this analysis, the global importance ranking of the features during the simulation for all participants, i.e., which pairs of events/physiological markers were the most predictive among all the participants, after excluding 'explosion' from the list of events is shown in Fig. 2. A similar result obtained by regrouping the features by the type of physiological markers (e.g., mean GSR, max HR, etc.) is illustrated on Fig. 3.

References

1. Magliacano, A., Fiorenza, S., Estraneo, A. & Trojano, L. Eye blink rate increases as a function of cognitive load during an auditory oddball paradigm. *Neurosci. Lett.* **736**, 135293, DOI: 10.1016/j.neulet.2020.135293 (2020).
2. Oh, J., Jeong, S.-Y. & Jeong, J. The timing and temporal patterns of eye blinking are dynamically modulated by attention. *Hum. movement science* **31**, DOI: 10.1016/j.humov.2012.06.003 (2012).
3. Huber, S., Martini, M. & Sachse, P. Patterns of eye blinks are modulated by auditory input in humans. *Cognition* **221**, 104982, DOI: 10.1016/j.cognition.2021.104982 (2022).
4. Säring, W. & Cramon, D. The acoustic blink reflex: Stimulus dependence, excitability and localizing value. *J. neurology* **224**, 243–52, DOI: 10.1007/BF00313287 (1981).

Tables

GAMESTART	FLASHBANG
AIRRAIDSIREN	EXPLOSION
M41	MP5
MRPEANUTBUTTER Activated	MRPEANUTBUTTER deactivated
ABRAMS Activated	
ABRAMS Entered INNERRADIUS	ABRAMS Left INNERRADIUS
ABRAMS Entered MAXIMUMATTENUATION	ABRAMS Left MAXIMUMATTENUATION
COPCOPTER Activated	COPCOPTER Fired
COPCOPTER Entered MAXIMUMATTENUATION	COPCOPTER Left MAXIMUMATTENUATION
COPCOPTER Entered INNERRADIUS	COPCOPTER Left INNERRADIUS
DUMMYTANK Activated	
DUMMYTANK Entered MAXIMUMATTENUATION	DUMMYTANK Left MAXIMUMATTENUATION
DUMMYTANK Entered INNERRADIUS	DUMMYTANK Left INNERRADIUS
PATRIOT Activated	
PATRIOT Entered MAXIMUMATTENUATION	PATRIOT Left MAXIMUMATTENUATION
PATRIOT Entered INNERRADIUS	PATRIOT Left INNERRADIUS
PUMA Activated	
PUMA Entered MAXIMUMATTENUATION	PUMA Left MAXIMUMATTENUATION
PUMA Entered INNERRADIUS	PUMA Left INNERRADIUS

Table 1. List of events occurring during the high-stress military-related condition. There were different types of events, which could involve visual and/or sound stimuli. 'M41', 'ABRAMS' 'DUMMYTANK' and 'PUMA' are military tanks, 'MP5' is a machine gun, 'COPCOPTER' is a helicopter, 'PATRIOT' is a military truck (HMMWV) and 'MRPEANUTBUTTER' is a barking dog. For most of the events, five types of statuses were possible during the simulation.
- 'Activated': The participant triggered the event, and the object (e.g., helicopter or tank) started moving.
- 'Entered MAXIMUMATTENUATION': The participant is close enough where sound levels are at a maximum.
- 'Left MAXIMUMATTENUATION': The participant is no longer close enough to hear the object at maximum sound levels.
- 'Entered INNERRADIUS': Due to movement of the object and/or the participant, the participant is getting closer (or has entered a specified radius) to the object.
- 'Left INNERRADIUS': Due to movement of the object and/or the participant, the participant is moving farther away (or has left a specified radius) from the object.

Figures

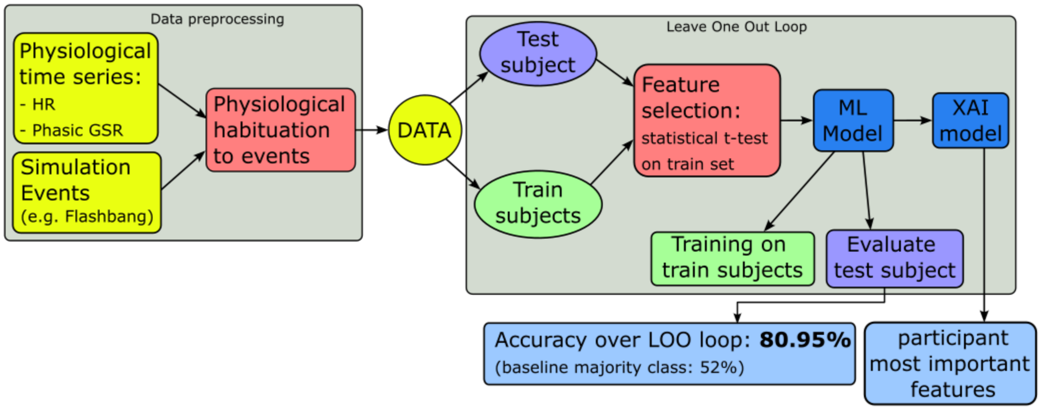

Figure 1. An overview of the pipeline of the entire algorithm in this work. The left part shows the data preparation and preprocessing, where habituation features were computed from the events and the physiological time series. The second part consists of the ML training and testing performed via a leave-one-out validation scheme to avoid data leakage between the training and test participants, and the ML explainability algorithm (XAI) was plugged into the ML model to obtain insight into the output prediction. This explainability output was further aggregated with the participant's habituation features to give the participant a global habituation response during the simulation.

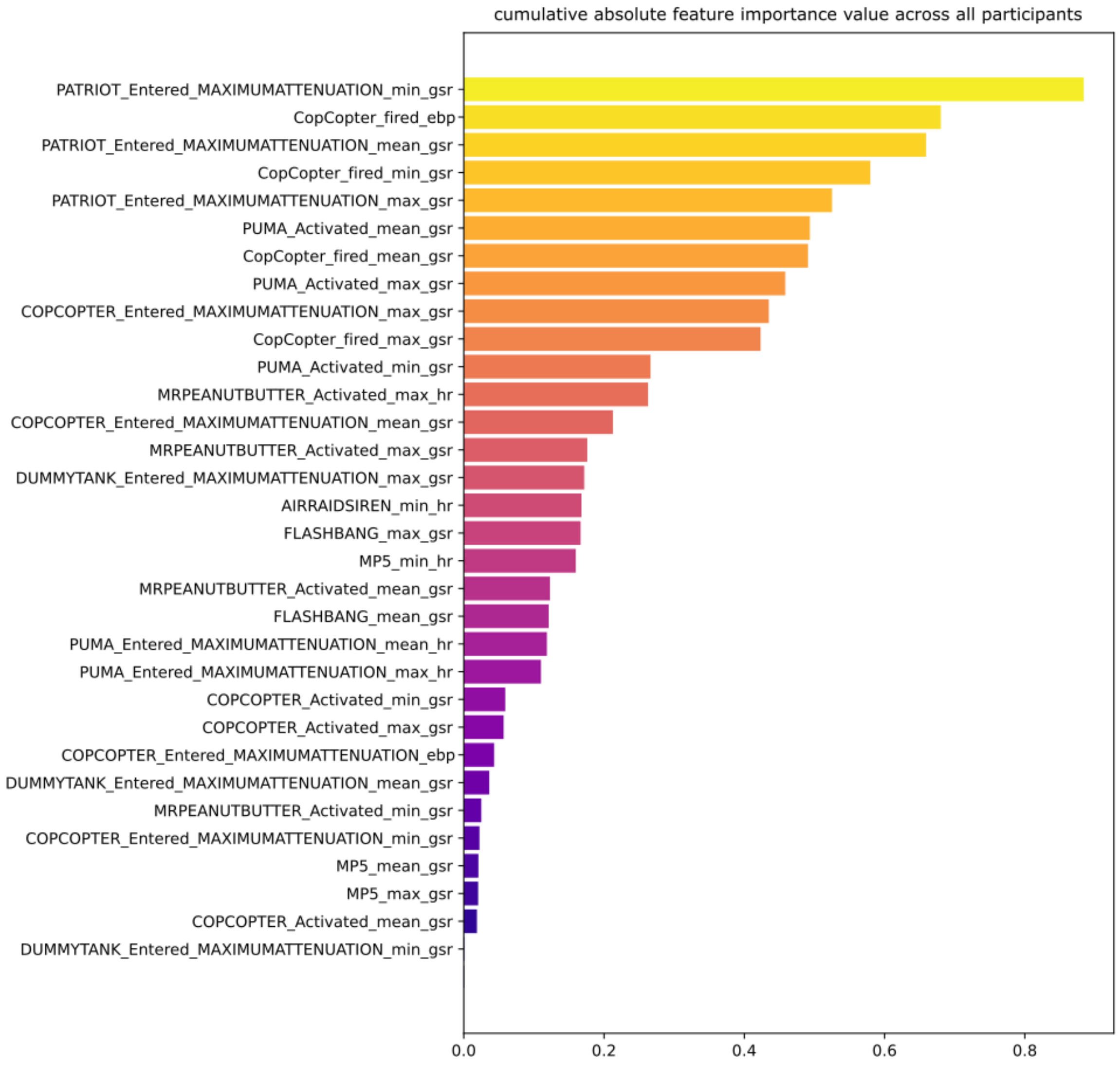

Figure 2. Ranking of the cumulative absolute importance value of the features during the simulation for all participants after excluding 'explosion' from the list of events. For each feature composed of an event/physiological marker pair, we summed the absolute importance value determined by SHAP for each test participant. The results provide an overview of the most important features for predicting PTSD or non-PTSD status across all participants and, therefore, which events are the most stressful, as well as which physiological features were the most related to these stress-inducing stimuli.

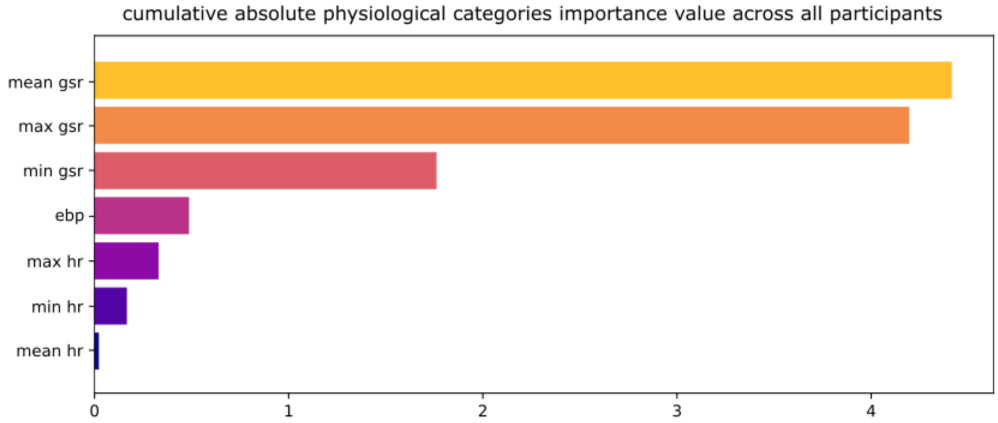

Figure 3. Ranking of the cumulative absolute importance value of the physiological features during the simulation for all participants after excluding ‘explosion’ from the list of events. This result was obtained from Figure 2 by grouping features of the same physiological category (e.g., max GSR, EBP, etc.). The results provide an overview of which physiological features aare the most predictive of PTSD or non-PTSD status across all participants.
